# Fingerpad‐Inspired Multimodal Electronic Skin for Material Discrimination and Texture Recognition

**DOI:** 10.1002/advs.202002606

**Published:** 2021-02-08

**Authors:** Giwon Lee, Jong Hyun Son, Siyoung Lee, Seong Won Kim, Daegun Kim, Nguyen Ngan Nguyen, Seung Goo Lee, Kilwon Cho

**Affiliations:** ^1^ Department of Chemical Engineering Pohang University of Science and Technology Pohang 37673 Korea; ^2^ Department of Chemistry University of Ulsan Ulsan 44 610 Korea

**Keywords:** biomimetic materials, electronic skin, sensors, stretchable electronics

## Abstract

Human skin plays a critical role in a person communicating with his or her environment through diverse activities such as touching or deforming an object. Various electronic skin (E‐skin) devices have been developed that show functional or geometrical superiority to human skin. However, research into stretchable E‐skin that can simultaneously distinguish materials and textures has not been established yet. Here, the first approach to achieving a stretchable multimodal device is reported, that operates on the basis of various electrical properties of piezoelectricity, triboelectricity, and piezoresistivity and that exceeds the capabilities of human tactile perception. The prepared E‐skin is composed of a wrinkle‐patterned silicon elastomer, hybrid nanomaterials of silver nanowires and zinc oxide nanowires, and a thin elastomeric dielectric layer covering the hybrid nanomaterials, where the dielectric layer exhibits high surface roughness mimicking human fingerprints. This versatile device can identify and distinguish not only mechanical stress from a single stimulus such as pressure, tensile strain, or vibration but also that from a combination of multiple stimuli. With simultaneous sensing and analysis of the integrated stimuli, the approach enables material discrimination and texture recognition for a biomimetic prosthesis when the multifunctional E‐skin is applied to a robotic hand.

## Graphical abstract

Fingerpad‐inspired multimodal electronic skin (E‐skin) is developed by mimicking functional and geometrical properties of a human finger. This multifunctional device can identify and distinguish not only mechanical stress from a single stimulus such as pressure, tensile strain, or vibration but also that to a combination of multiple stimuli, enabling material discrimination and texture recognition for a biomimetic prosthesis.

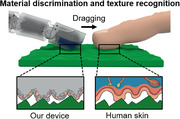

Skin is the largest organ of the human body. It communicates with a person's environment by facilitating mechanical interactions such as pressure, vibration, and tensile strain associated with skin deformation in daily activities.^[^ ^1^, ^2^ ^]^ In particular, somatosensory of shear (dragging) force detection for human skin enables the detection of more complex and combined stimuli, which can aid in the recognition of the texture properties (e.g., roughness, stickiness, and stiffness) of touched objects.^[^ ^3^, ^4^, ^5^, ^6^ ^]^ In this tactile perception, skin friction is regarded as a force that resists the skin motion relative to other surfaces and is a key factor for detecting shear force by activating mechanoreceptors in human skin.^[^ ^7^ ^]^ During dragging processes, in particular, the fingerprint on the skin enhances adhesion and friction of the fingerpad, acting as a magnifying layer, and thereby elevating the surface strain with surface deformation.^[^ ^8^ ^]^ However, the aforementioned mechanoreceptors alone do not enable sufficient perception of material substances and texture. To identify and classify diverse materials (metal, polymer, or ceramic) and textures, humans use not only their skin for tactile sensing, but also other sensory organs such as their eyes.^[^ ^9^ ^]^ Therefore, the integration of multiple modalities (touch and vision) and a complex process for discriminating materials and textures is compulsory.

With the rapid advancement of human‐friendly electronics, artificial nerves, and biomimetic prosthetics, electronic skin (E‐skin) will play an important role in the advancement toward next‐generation electronic technology, which transduces various mechanical signals such as pressure,^[^ ^10^, ^11^, ^12^ ^]^ vibration,^[^ ^13^, ^14^ ^]^ strain (stretching),^[^ ^15^, ^16^ ^]^ and shear force into electrical data as the human skin does.^[^ ^17^, ^18^ ^]^ Wearable and attachable E‐skin is frequently used for artificial intelligence,^[^ ^19^ ^]^ health monitoring of medical diagnostics,^[^ ^20^, ^21^, ^22^, ^23^ ^]^ and human machine interfaces for robotics.^[^ ^24^, ^25^, ^26^, ^27^, ^28^, ^29^, ^30^, ^31^ ^]^ Advanced progress in the development of E‐skin has been achieved through understanding, mimicking, and overcoming the ability of human skin especially including tactile perception.^[^ ^32^, ^33^, ^34^, ^35^, ^36^, ^37^ ^]^ Among the developed E‐skins, a few devices offer unique advantages with respect to texture recognition when compared with conventional E‐skin devices, including the ability to detect multiple tactile stimuli. Because most sensors operate under a single working mechanism, ferroelectricity,^[^ ^32^ ^]^ or piezoresistivity,^[^ ^33^ ^]^ they can only distinguish the roughness of each material surface. More recently, Choi et al. demonstrated a multimodal sensor that selectively responds to various stimuli and can differentiate among them.^[^ ^34^ ^]^ Because of its unique design and combination of triboelectric and piezoresistive mechanisms, their sensor could discriminate static pressure and vibration through analysis of the signals acquired via each mechanism. However, their device is also limited to the precise detection of minute surface roughness, being unable to identify and classify the material or its detailed textural properties. Thus, the sensors developed in previous studies cannot intrinsically solve the problems of material discrimination and texture recognition. The development of complete tactile perception through simultaneous sensing of pressure, tensile strain, and vibration using hybrid working mechanisms is critical to the development of an advanced E‐skin with capabilities that exceed the tactile perception ability of humans.

Here, we present a novel approach to detecting pressure, tensile strain, and vibration via hybrid triboelectric, piezoelectric, and piezoresistive operating mechanisms even though the stimuli are applied simultaneously. Moreover, in mimicry of the fingerprint morphology and tactile perception ability of human finger skin, our E‐skin device can discriminate among the aforementioned mechanical stimuli when shear force is applied during the dragging event. Therefore, our device can identify and classify materials such as metals, polymers, and ceramics and detailed texture information, demonstrating a new concept for the development of tactile‐perceptive E‐skin for advanced applications such as biomimetic prostheses and soft robotics which has never been approached before.

**Figure** **1**a schematically illustrates the tactile perception mechanisms of mechanoreceptors in the human somatosensory system when a human finger is dragged on a rough surface. The shear force induces three steps of mechanical stimuli, pressure, tensile strain, and vibration.^[^ ^38^ ^]^ First, as a finger approaches to a rough object, contact between the skin surface and the object induces pressure, which is detected by Merkel cells (the density of receptors: 70 cm^−2^, sensing range: > 2 kP).^[^ ^1^ ^]^ In this process, the fingerprint on human skin increases skin friction and magnifies the surface strain.^[^ ^8^ ^]^ Second, when the finger starts to move, friction between the two surfaces evokes a resistance force opposite the direction of the movement because of the adhesion force.^[^ ^6^ ^]^ The moving object possesses two types of friction: Static and kinetic. Because of the adhesion, if the magnitude of the static friction exceeds that of kinetic friction, the skin surface adheres to the object, being deformed under tensile strain and stimulating the Meissner corpuscle (the density of receptors: 140 cm^−2^, sensing range: 10–200 Hz).^[^ ^1^ ^]^ Third, additional dragging force is applied to the skin, which results in movement of the finger, dragging phenomena, vibration of the adhered skin surface, and activation of the Pacinian corpuscle (the density of receptors: 21 cm^−2^, sensing range: 40–800 Hz)^[^ ^1^ ^]^ in the scenario where kinetic friction exceeds static friction. Thus, the shear force can be detected using three sensory organs for pressure (Merkel cell), tensile strain (Meissner corpuscle), and vibration (Pacinian corpuscle).^[^ ^4^ ^]^ With these functions of the sensory organs, humans can recognize detailed information related to surface texture.

**Figure 1:**
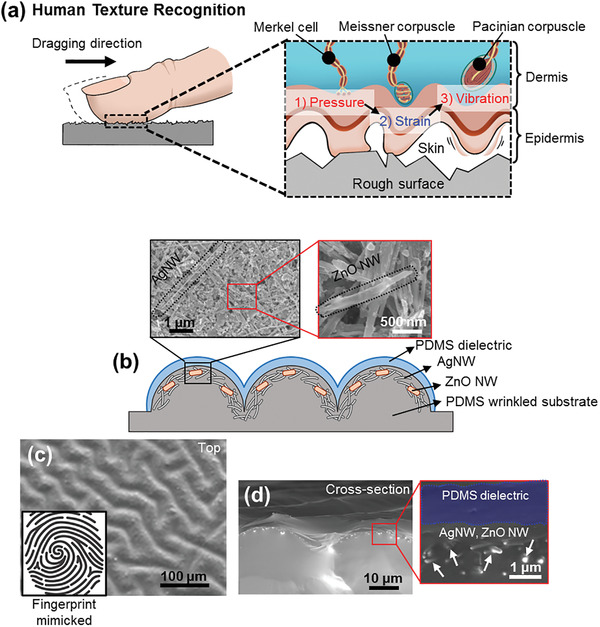
Concept and design of the multimodal mechanoelectric sensor. a) Schematic diagram of texture recognition mechanism with mechanoreceptors in human skin. b) Device structure composing of PDMS wrinkled substrate, hybrid nanomaterials including AgNW and ZnO NW, and thin PDMS dielectric layer. Insets are SEM images of the randomly mixed hybrid nanomaterials. c) OM image of microwrinkled and fingerprint mimicked E‐skin with top view. Inset: Shape of human fingerprint. d) Cross‐sectional SEM images of all PDMS‐based device, consisting of nanomaterials embedded in PDMS with microwrinkled structure.

To mimic the somatosensory organs in human skin, we fabricated a multimodal sensor based on hybrid properties of triboelectric, piezoelectric, and piezoresistive mechanisms. The prepared sensor comprises three parts: A microwrinkled polydimethylsiloxane (PDMS) substrate, a nanomaterial mixture of silver nanowires (AgNWs) and zinc oxide nanowires (ZnO NWs) for stretchable electronic functionality, and a thin PDMS dielectric layer coated along the wrinkle surface (Figure 1b). The dielectric PDMS layer is perfectly covered on the nanomaterials along the surface of PDMS wrinkles (amplitude: 15 µm, wavelength: 40 µm), which resembles the shape and function of human skin fingerprints (Figure 1c). The fabrication methods are summarized as three steps: Wrinkle fabrication, deposition of nanomaterials, and coating of a thin dielectric layer (Figure S1, Supporting Information). To fabricate the thin PDMS dielectric structure, PDMS was cured in an upside‐down position after being spray‐coated (Figures S3 and S4, Supporting Information). Because of the thin morphology of the dielectric layer, it does not flatten the microwrinkles of the substrate, maintaining the large surface area of the sensor (Figure 1d) and maximizing the electrical output performance of the E‐skin. In addition, this device exhibits robust mechanical stability under external stimuli because of the device structure of the all‐PDMS‐based system.

The sensor performance under a single stimulus of pressure, vibration, or dynamic/static strain is shown in **Figure** **2**. Each operation mechanism responds with specific mechanical stimuli such as triboelectricity for vibration, piezoelectricity for dynamic strain, and piezoresistivity for static pressure and strain. First, the triboelectric property arises from contact electrification, which is a key process for the charge separation activating at the interface of the two contact materials.^[^ ^39^ ^]^ The triboelectric voltage (*V*) is mainly proportional to three factors: The contact area (*S*) and distance (*x*) between the two objects, and the surface charge of the materials (*σ*). It is defined as

(1) $$V\;=\;a\left[S\right]\times \left[x\right]\times \left[\sigma \right]$$

where *a* is an empirical coefficient. The voltage generated from triboelectricity is investigated under increasing pressure. As shown in Figure 2a, the generated voltage signals increased as the pressure was increased from 10 to 90 kPa. We also investigated the effect of surface topologies on the triboelectricity. The triboelectric voltage generated with microstructured, 50% stretched microstructured, and flat dielectrics was also measured under repeated cycling of pressure between 10 and 90 kPa to characterize the contact area effect resulting from the triboelectric property (Figure 2b). The surface roughness of the dielectric layer is a key factor in the generation of the triboelectric voltage. Therefore, with increasing surface area and pressure, the magnitude of the change of the contact area increases, followed by an increase in the generated voltage.

**Figure 2:**
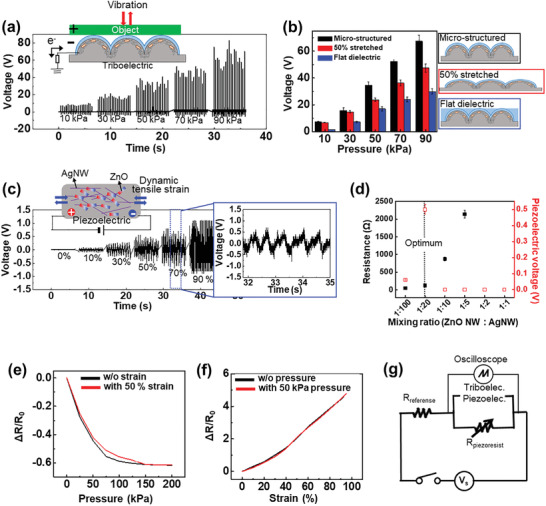
Electrical performances of single‐stimulus detection. a) Voltage generation with triboelectric property under repeated pressure up to 90 kPa. Inset: Mechanism of triboelectric under vibration. b) Triboelectric voltage deviation with pressure change for different geometries of the dielectric on E‐skin device (*n* = 5). c) Voltage change under stretching and releasing from 0% to 90% strain with schematic diagram of piezoelectric mechanism. Inset: Enlarged graph from 32  to 35 s. d) Piezoelectric voltage with various mixing ratios of the nanowires (*n* = 5). e,f) Normalized resistances (Δ*R*/*R*_0_) versus e) pressure with/without 50% of stretching and f) strain with/without 50 kPa of pressure. g) Circuit diagram of the prepared E‐skin device

As shown in Figure 2c, with stretching‐releasing under tensile strain, the ZnO NWs in the PDMS deform, resulting in displacement of the cations and anions, which leads to an asymmetrical charge distribution and induces a dipole moment in nanomaterial, followed by the generation of electrical voltage via the piezoelectric properties resulting from the asymmetric electric polarization.^[^ ^40^ ^]^ The overall change in the dipole moment produces an electrical potential difference in the device, causing a piezoelectric voltage. As the tensile strain increases from 0% to 90%, the generated voltage increases from 0.15 to 1.2 V. The mixing ratio of the nanowires was optimized for the maximum value of a piezoelectric voltage under connected current paths between AgNWs (Figure 2d). However, the presence of conductors between piezoelectric materials, compared to fully insulating state, can slightly inhibit the piezoelectric performance of our device (Figure S5, Supporting Information). We also measured the piezoelectric voltage under repeated pressures at 1 and 2 Hz (Figure S6, Supporting Information). Moreover, to detect the static state of pressure and strain, we used the piezoresistive property, which originates from a variation of the AgNW percolation and results in a resistance difference. The resistance changes for pressure with or without 50% tensile strain were measured over the pressure range from 0 to 200 kPa (Figure 2e). As the pressure increases, the percolation among AgNWs increases, followed by a decrease of the resistance. The resistance also increases when a static tensile strain is applied up to 100% mechanical strain under the condition with or without 50 kPa of pressure (Figure 2f). As a result, the prepared sensor exhibits versatile ability to detect both dynamic and static states of pressure and tensile strain and even discriminates between different mechanical stimuli. Figure 2g shows a circuit diagram of this multimodal sensor. The signals from the triboelectric/piezoelectric mechanisms and the piezoresistive mechanism cannot be simultaneously measured. Therefore, we measured signals from those mechanisms separately. For triboelectric and piezoelectric mechanisms, we conducted the measurements under the open circuit conditions. In other words, the two electrodes of the device are connected directly to an oscilloscope to collect the output voltage changes. However, in case of the piezoresistive mechanism, we needed to measure under a short circuit, so that we additionally connected a reference resistance and a power source to characterize the voltage change of the device under voltage bias state. The performance of this sensor was characterized using only an oscilloscope, which is easy to acquire and whose signals are easily analyzed under a constant overall voltage.

**Figure** **3** shows the performance of the multimodal sensor under simultaneous and sequential innervation. The sensor can discriminate each stimulus from the generated voltage waveforms because of its various response signal mechanisms (e.g., the triboelectric, piezoelectric, and piezoresistive mechanisms). The presence of pressure (40 kPa) increases the percolation density of AgNWs and decreases the resistance, accompanied by decrease of the voltage (Figure 3a). When the vibration was additionally applied under this condition, triboelectric voltage was generated. After the pressure was removed, the voltage signal recovered. Figure 3b shows the voltage‐time curves corresponding to the sequential application of multiple stimuli of dynamic strain and vibration. When the device is repeatedly stretched and released with 50% strain, the piezoelectric signal is generated in response to the mechanical stress. In addition, the vibration in the stretching cycle generates both piezoelectric and triboelectric signals, as evident form the peak shape of the voltage change. Even in the simultaneous application of the multiple stimuli, the triboelectric voltage waveform can be distinguished from the piezoelectric voltage waveform (Figure S7, Supporting Information). The case of dual activations with a static pressure of 200 kPa and 80% static tensile strain is shown in Figure 3c. As the pressure is applied, the resistance decreases as the percolation ratio between the AgNWs increases, followed by a decrease in the voltage of the device under the constant overall voltage condition (Figure 2f). In this situation, an increase in strain increases the voltage in response to the increase in resistance. In Figure 3a,c, PDMS‐coated objects were used to avoid generating the triboelectric voltage when the static pressure was applied. With this method, we could discriminate the output signals between piezoresistivity for static pressure and triboelectricity for vibration. Therefore, the data obtained from multimodal sensing are perfectly fit through single stimulus detection and enable discrimination among different external stresses via the aforementioned operating mechanisms. The experimental setups for measurements are shown in the Supporting Information (Figure S2, Supporting Information).

**Figure 3:**
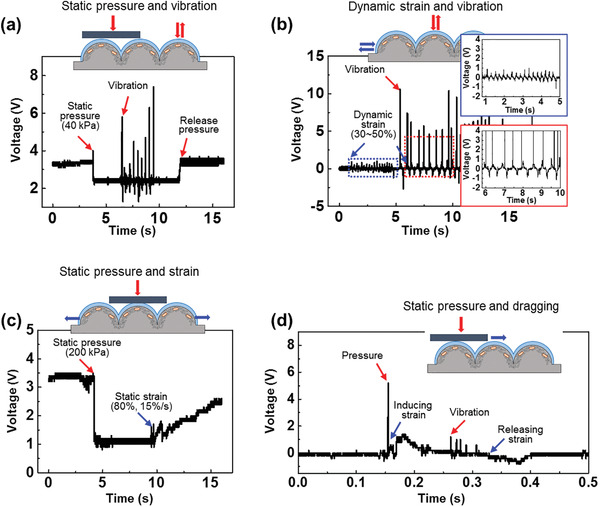
Electrical performances of multi‐stimuli detection. Voltage changes under sequential stimuli. (Insets: Experimental schemes for external stimuli) a) Pressure (40 kPa) and vibration, b) dynamic strain and vibration, c) pressure (200 kPa) and static strain (80%, 15% s^–1^), and d) pressure (10 kPa) and dragging (speed: 2.0 mm s^–1^), inducing shear force.

As shown in Figure 3d, we characterized the performance of the multimodal sensor in the detection of multiple stimuli of static pressure/strain and vibration while dragging a metallic object across the sensor. Human skin can detect shear force with three sensory organs for pressure (Merkel cells), tensile strain (Meissner corpuscles), and vibration (Pacinian corpuscles).^[^ ^4^ ^]^ Similar to the function of the mechanoreceptors in human skin, our sensor can detect and discriminate the external stimuli during the dragging event. When an object is dragged across the sensor device, the triboelectric signal appears with contact between the sensor and the object surface. The contact between two objects induces friction force involving the contribution of adhesion (directly related to the area of contact and interfacial energy) and a deformation (affected by geometry and stiffness) factor.^[^ ^4^, ^7^ ^]^ The friction force equation is typically defined as *f*_*μ*_:

(2) $$f_{\mu }=f_{\mu }^{\mathrm{adh}}\;+f_{\mu }^{\mathrm{def}}$$

where $f_{\mu }^{\mathrm{adh}}$ and $f_{\mu }^{\mathrm{def}}$ are the adhesion and deformation‐induced components of the friction force, respectively. In greater detail, the adhesion‐induced friction force is defined as:

(3) $$f_{\mu }^{\mathrm{adh}}=\;\tau A$$

where *A* is the area of contact and *τ* is the interfacial strength. Moreover, numerous researchers have found that interfacial adhesion is the main contributor to friction force, whereas deformation‐induced friction is a minor contribution when the two objects are not easily plastic‐ or viscoelastic‐deformed.^[^ ^41^, ^42^, ^43^ ^]^ Adhesion between two surfaces induces static friction, which results in tensile strain that resists the movement of the object and activates the piezoelectric signal with elongation of the E‐skin device. As more force is applied to move the object, the vibration and triboelectric signals emerge via the dragging (stick‐slip) phenomenon between two surfaces. Finally, tensile strain is removed when the shear force ends. The changes in the chemical and geometrical dielectric structures of the devices strongly affect the shear force recognition performance (Figure S8, Supporting Information). A lower surface energy and surface area lead to weaker adhesion between the contact materials, which results in a decrease in the voltage signal and inhibits the detection of the shear force.

In **Figure** **4**, we demonstrate the superior performance of our device in recognizing material and texture information about the contacted surface. Figure 4a illustrates an application of material and texture recognition for robotic prosthesis that exceeds human tactile perception. The device placed on the robotic finger can distinguish among contact materials such as a metal, polymer, and ceramic and can recognize detailed textures of its surface by touching and rubbing the object. As shown in Figure 4b, we attached the E‐skin to a robotic hand and dragged the device across various materials (Figures S9–S11, Supporting Information), similar to the function of the human tactile perception mechanism. Each material possesses distinct properties of roughness, surface energy, and surface charge density. In Figure 4c, we conducted a sweep on human skin with the E‐skin device at constant pressure (≈10 kPa) and speed (20 mm s^–1^). The dragging of the sensor generates time‐dependent electrical output signals through interaction between the surfaces of the sensor and the object. The output data indicate that a triboelectric peak signal (A) was obtained from contact between the sensor and object, followed by a piezoelectric signal (B) of strain from the adhesion force and a triboelectric voltage of vibration from detachment. The human skin exhibits high surface‐charge potential, generating a strong triboelectric signal under contact because of the stratum corneum lipid matrix in the skin's outer layer.^[^ ^44^, ^45^ ^]^ Moreover, the human skin has a low surface energy and roughness, which decreases the adhesion force between the object and the device and evokes a weaker piezoelectric signal originating from deformation of the E‐skin. A fast Fourier transform (FFT) was used to acquire the vibration frequency response, and the resultant quantitative information about the adhesion force was analyzed (Figure 4d). The response performance was initially characterized on the basis of the vibration induced by the dragging motion. This motion comprises contact and detachment between the device and object under constant pressure and speed. As the adhesion force between two surfaces increases, in the case of nanostructured gold (root‐mean squared surface roughness: 91.9 nm), the amplitude of vibration becomes large after detachment of the interface during the dragging motion (Figure 4e,f). Therefore, the amplitude obtained by FFT analysis of the output voltage signal mainly depends on the adhesion force, resulting from the roughness, surface energy, or modulus of the contacted object.

**Figure 4:**
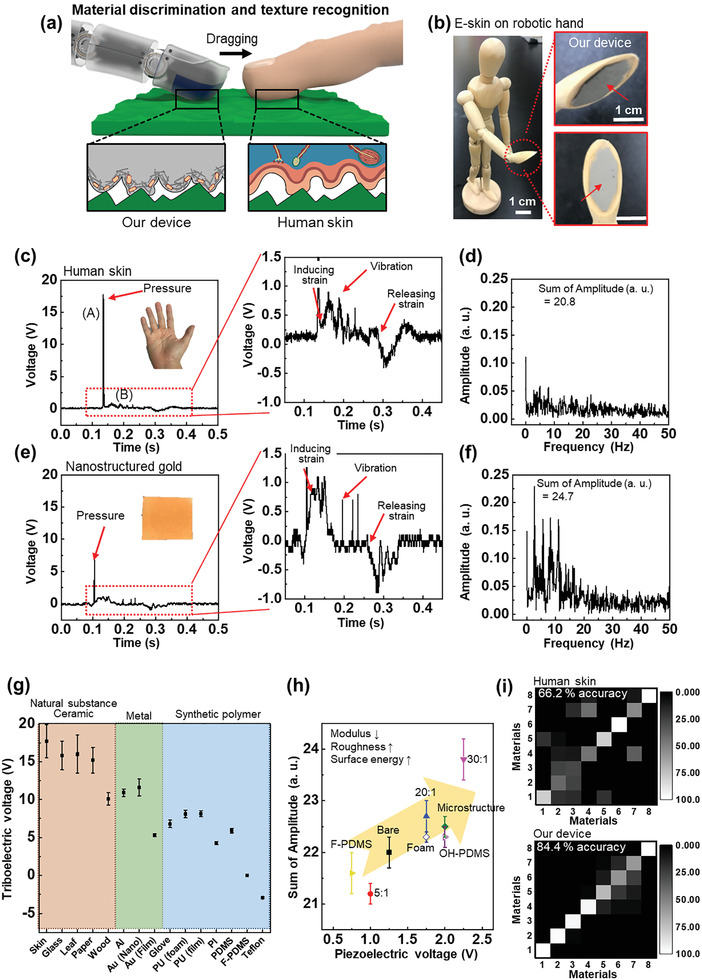
Demonstrations for material discrimination and texture recognition. a) Schematic diagram of material/texture recognition for robotic prosthesis. b) Photograph of the E‐skin on robotic hand. Inset is a magnified photo of the robot hand, attaching the E‐skin on it. c,e) Material and texture‐dependent electrical changes and d,f) frequency response signal while dragging the device on human skin and nanostructured gold, respectively. g) Triboelectric voltage with various contact materials for recognizing the material substances (*n* = 10). h) Piezoelectric voltage versus sum of amplitude for frequency to distinguish the various PDMS samples with different textures including modulus (mixing ratio of pre‐polymer and crosslinker, 5:1, 10:1, 20:1, 30:1), roughness (2D microstructured and 3D foam), and surface energy (F‐PDMS and OH‐PDMS) (*n* = 10). i) Confusion matrix for classification in PDMSs with the eight different textures, mentioned before, corresponding from material 1 to 8, by using human skin and our device. The matrix, visualizing the difference of color, shows the probability for classifying one PDMS from the eight PDMSs. The accuracy of total classification for eight PDMSs is 62.2 ± 0.5% for human skin and 84.4 ± 0.4% for our device (*n* = 100, p < 0.01).

To identify and classify the electric signal of the shear force induced by dragging our sensor on various materials and complex surface textures, we categorized the electrical data obtained from each object on the basis of the maximum triboelectric voltage/piezoelectric voltage and the sum of the amplitude of the FFT frequency. (The raw data for each material are presented in Figures S12 and S13, Supporting Information) Figure 4g shows that our sensor can distinguish among materials such as a natural substance, ceramic, metal, and synthetic polymer by differentiating the triboelectric voltage values. The generated triboelectric voltage is related to the surface potential of each contacted material (Figure S14, Supporting Information). Moreover, the surface energy, roughness, and modulus can be also detected by comparing the piezoelectric voltage and the sum of the amplitude of the frequency values with those for other objects. To fabricate various textures of contacted objects, the mixing ratio of curing agent, the surface geometry, and the surface energy of PDMS were tuned. As shown in Figure 4h, when the surface energy and roughness increase and the modulus decreases, both the piezoelectric voltage and the sum of the amplitude for frequency increase because of an increase of the adhesion force between our sensor and the contacted object. To analyze and distinguish the output signals from the E‐skin device, we implemented a confusion matrix.^[^ ^33^, ^34^ ^]^ Eight different PDMS samples, as previously mentioned, were tested to discriminate the surface texture information. The electrical signals were acquired by dragging the samples with our sensor, followed by FFT. Using this method, we compared each FFT signal from PDMS samples with various textures. As shown in Figure 4i, our E‐skin device successfully classified the various PDMSs with 84.4 ± 0.4% classification accuracy using only the texture information gathered by our sensor; by comparison, the classification accuracy of human skin was 62.2 ± 0.5%. During the dragging motion for material discrimination and texture recognition, we only used triboelectric and piezoelectric mechanisms to detect the dynamic responses. With the triboelectric mechanism, we can acquire the material information through the contact between our device and the contacted object, followed by the generation of triboelectric voltage. In the case of a piezoelectric mechanism, the texture information can be obtained by comparing the output voltage originated from the different adhesion forces between our device and the object. These results imply that our versatile E‐skin can be used in robotic prosthesis application as a sensory organ with tactile perception ability similar to or better than that of humans.^[^ ^46^ ^]^

In conclusion, we developed a human skin inspired, stimuli‐discriminable, and multimodal E‐skin that can recognize a material and surface texture on the basis of shear force consisting of pressure, tensile strain, and vibration. The novel device structure mimicking human fingerprint was demonstrated, where the output signal under external mechanical stimuli was maximized. In addition, we used two nanomaterials such as AgNWs and ZnO NWs, resulting not only in hybrid triboelectric, piezoelectric, and piezoresistive properties but also in stimuli‐discriminable functionality by fitting each signal to each stimulus. Given these outstanding abilities of our E‐skin, we demonstrated for the first time a device that can perceive various material substances and surface textures such as roughness, surface energy, and surface charge density with effectiveness beyond the capability of human tactile perception, thereby providing a unique platform for application in humanoid robotics, wearable sensors, and biomimetic prosthetic systems.

## Conflict of Interest

The authors declare no conflict of interest.

## Supporting information

Supporting Information

## Data Availability

Data sharing is not applicable to this article as no new data were created or analyzed in this study.
