# Supplementary material for: Fingerpad‐Inspired Multimodal Electronic Skin for Material Discrimination and Texture Recognition
Source: Adv Sci (Weinh). 2021 Feb 8;8(9):2002606. doi: 10.1002/advs.202002606 (PMC8097346; doi:10.1002/advs.202002606)
Supplement: Supplementary file 1 — Supporting Information [file ADVS-8-2002606-s001.pdf]

© 2021 Wiley-VCH GmbH

ADVANCED SCIENCE
Open Access

Supporting Information

for *Adv. Sci.,* DOI: 10.1002/advs.202002606

Fingerpad-Inspired Multimodal Electronic Skin for Material Discrimination and Texture Recognition

Giwon Lee, Jong Hyun Son, Siyoung Lee, Seong Won Kim, Daegun Kim, Nguyen Ngan Nguyen, Seung Goo Lee, and Kilwon Cho**

Supporting Information

Fingerpad-Inspired Multimodal Electronic Skin for Material Discrimination and Texture Recognition

Giwon Lee, Jong Hyun Son, Siyoung Lee, Seong Won Kim, Daegun Kim, Nguyen Ngan Nguyen, Seung Goo Lee*, and Kilwon Cho*

Department of Chemical Engineering,
Pohang University of Science and Technology, Pohang, 37673 (Korea)

Department of Chemistry,
University of Ulsan, Ulsan, 44610 (Korea)

I. Supplementary Methods

I.1. Preparation of multimodal sensor

The PDMS film was prepared with a PDMS prepolymer and a curing agent (Sylgard 184, Dow Corning, weight ratio 20:1). The square (30 mm × 30 mm) PDMS substrate (~0.5 mm thick) was biaxially stretched, then exposed to UVO (Jelight 42-220, 28 mW cm^{-2}) for 60 min.[1] The pre-strain was simultaneously taken away from every axial direction, inducing a random wrinkled PDMS substrate (**Figure S1**). Mixed solution of a silver nanowire (AgNW, 0.02 g/ml in water base, ACS materials) and zinc oxide nanowire (ZnO NW, 0.001 g/ml in water base, Sigma Aldrich) was dropped on the wrinkled substrate. After evaporation of the droplets, the hybrid nanomaterials were uniformly deposited on the PDMS substrate. The precured PDMS (20:1) was diluted in hexane with ratio of 3:1. The solution was spray-coated on the layer of nanomaterials, followed by PDMS curing process. When the coated substrate was placed on upside-down position during the curing process, the PDMS dielectric layer can be covered along the surface of the nanomaterials layer.

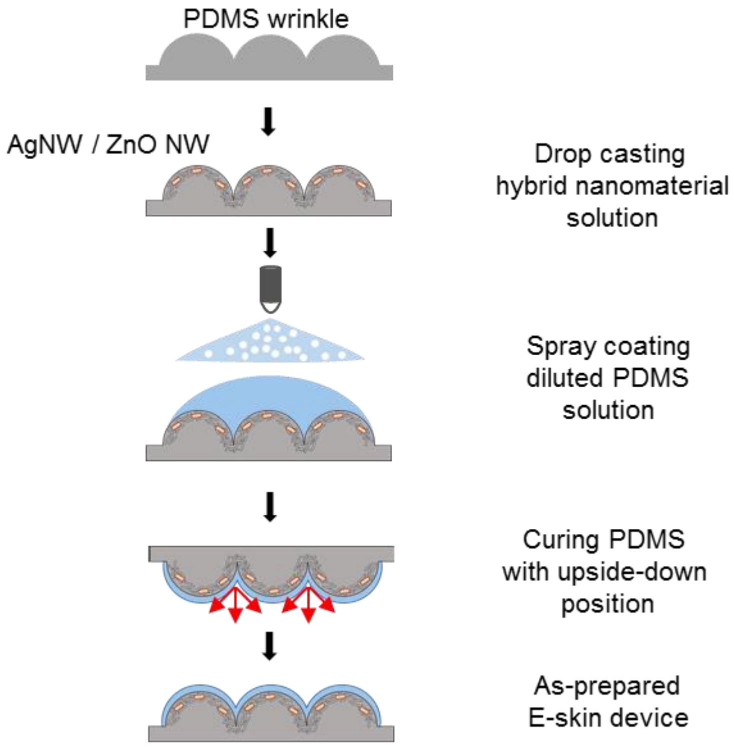

Figure S1. Schematic diagram of the fabrication processes

I.2. Sample Characterization

The sample morphologies were characterized by using field emission scanning electron microscopy (FESEM) (Hitachi S-4200), and optical microscopy (OM) (Axioplan Zeiss). The electrical properties of the strain sensor and pressure sensor, current-voltage (*I*–*V*) curves were measured using a Keithley S4200 instrument. The electrical voltage outputs were acquired by an oscilloscope (Tektronix, TBS2102). We measured signals from three operating mechanisms such as triboelectricity, piezoelectricity and piezoresistivity to detect different types of stimuli. For triboelectric and piezoelectric mechanisms, we conducted the measurements under open circuit conditions, which means that two electrodes of the device are connected directly to an oscilloscope to collect the output voltage changes. However, in case of the piezoresistive mechanism, we needed to measure the signals under a short circuit,

so we additionally connected a reference resistance and a power source to characterize the voltage change of the device under voltage bias state. The tensile strain was applied by using a motion controller with a speed of 20 mm/s (Autonics Co., PMC-2HS 2axis Motion controller). To measure a degree of applied pressure, a mechanized z-axis stage (Future Science, 0.1 μm resolution) and a force gauge (Mark 10) were used (**Figure S2**). The surface potential distribution and contact potential difference (CPD, V_{CPD}) was collected by Kelvin probe force microscope (KPFM) (Multi-Mode 8, Bruker) with a Pt coated tip. Accuracy of tactile classification was acquired by using the Confusion matrix.[2] All procedures were approved by the Research Ethics Committee of Pohang University of Science and Technology in South Korea (PIRB-2020-E017). Written informed consents were obtained from all subjects.

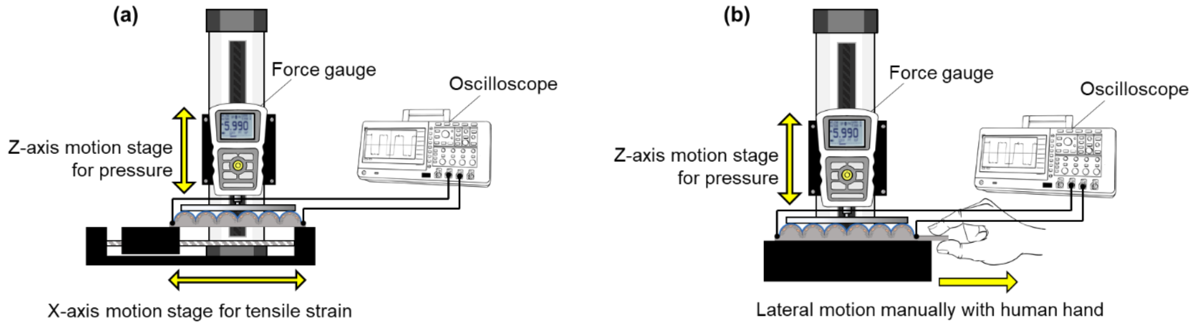

Figure S2. Schematic diagrams for measurement setups of pressure/tensile strain (a) and dragging motion (b)**.**

I.3. Statistical Analysis

Most of data were presented as mean ± standard deviation (SD), obtained from at least five independent experiments. Two-way analysis of variance (ANOVA) and Confusion matrix

were used for statistical analysis. The statistical values of $p < 0.01$ were shown, indicating the reliability of this statistical method. The numbers of sample size (n) for each experiment were indicated in the figure legends. The statistical analysis was carried out using IBM SPSS® software**.**

II. Mechanisms of Thin Dielectric Formation along the PDMS Wrinkled.

The key point of the fabrication processes of this multimodal sensor is curing condition. There are three factors of curing PDMS after the spray coating of diluted PDMS solution including temperature, time, and placed position. Especially, to successfully manipulate the thin dielectric along the surface of wrinkled PDMS substrate, the sample placed position during the curing is the most important part to adjust various applied fluidic forces in PDMS solution right after spray-coating step (**Figure S3**). After the coating, gravitational, capillary, and viscous forces are dominant to reduce the roughness substrate with the PDMS solution.[3] In this mechanism, rough surface can be flattened by filling the trough part of the PDMS wrinkle under normal curing positon. For the case of upside-down curing position, however, the gravitational force is opposite to the previous case by inhibiting to fill the roughness of the wrinkled substrate, which results in coating thin film morphology of the PDMS dielectric layer. This thin shape of PDMS dielectric maintains large surface area of the sensor substrate, leading to enhanced electrical performance of the device (**Figure S4**).

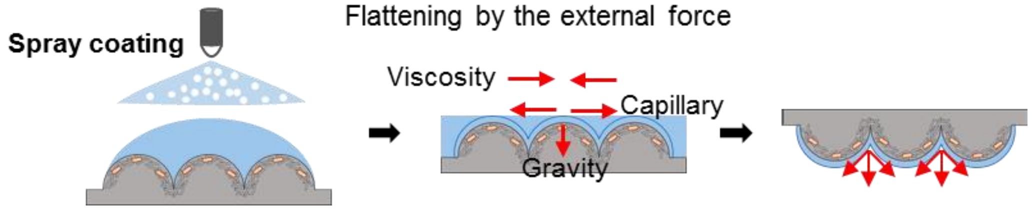

Figure S3. Schematic diagram of spray coating and curing PDMS.

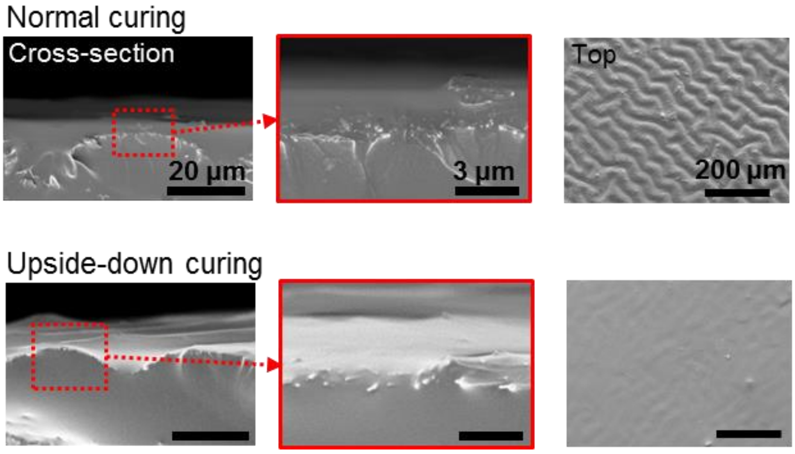

Figure S4. SEM images of PDMS dielectric coated morphologies with normal curing and upside-down curing.

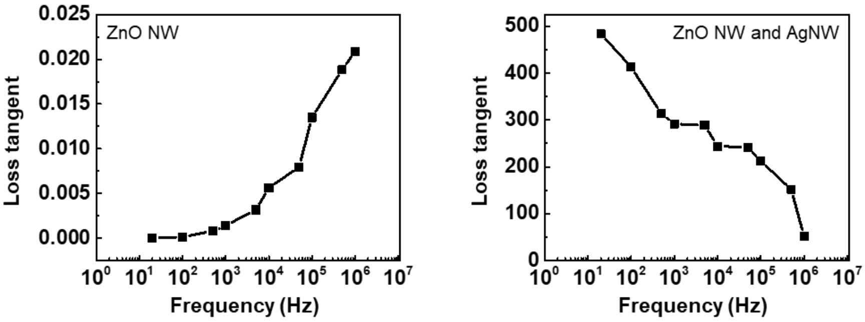

Figure S5. Loss tangent measurement with frequency variation for two cases including only ZnO NW and ZnO NW/AgNW.

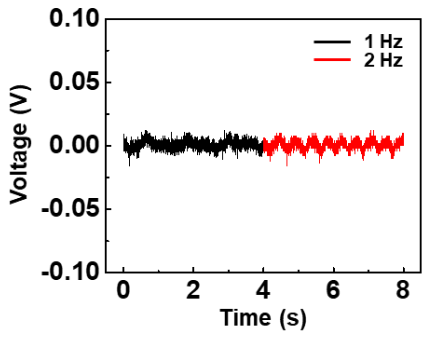

Figure S6. Voltage changes under repeated pressures at 1 Hz and 2 Hz.

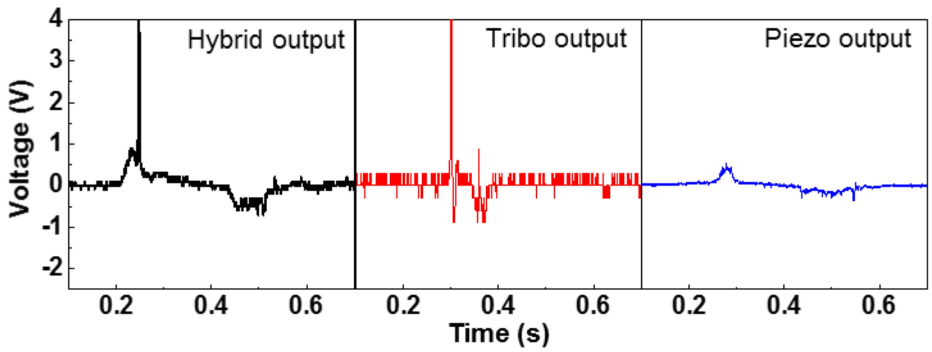

Figure S7. Voltage waveforms consisting of triboelectric and piezoelectric voltages under the simultaneous stimuli.

III. Mechanisms and Importance of Friction Force during Dragging Events.

As shown in Figure S7, the shape and time scale of output voltages between triboelectric and piezoelectric mechanisms are different from each other. We can decouple the dragging motion into two step, touch and lateral movement. When our device touches the surface of a detecting object, the triboelectric voltage with a sharp narrow peak is generated by contact electrification. After that, the piezoelectric voltage peak with a sinusoidal wave appears due

to the stick-slip behavior (adhesion) between the device and the object under the lateral movement. At the end, the detachment produces the negative signal of piezoelectric voltage.

The friction involves the effective factors of both an adhesion and a deformation component.[4] The adhesion component is directly connected to the notion of real area and surface energy of contact (sum of micro-structured contact areas). The deformation component is associated with the geometry and deformation of asperities that resist the relative motion of the contacting surfaces. In solid mechanics, surface roughness (i.e. geometric characteristics of surface topography at a small scale) of materials is main contributor to friction.

III. 1. Dragging Detection with the Modification of Chemical and Physical Morphology of PDMS Dielectric.

We also changed the dielectric morphology of the E-skin with chemical and physical conditions (**Figure S8**). When the dielectric is tuned with trichloro(1H, 1H, 2H, 2H-perfluorooctyl)silane, the surface is replaced with Fluorine atom (F), which results in increasing electronegativity of the surface. In this reason, more electrons are collected on the dielectric surface, followed by increasing surface charge potential and generating more output voltage from triboelectricity. In case of adhesion, however, lower surface energy because of the fluorine atom lead to lower adhesion force, which affects to reduce friction force and output voltage of strain and vibration from dragging force. When the dielectric is flat with a fluorinated surface, because of lower surface area, the friction force and surface charge density apparently became lower. These result in poor voltage signal from the shear force. Therefore, the E-skin with thin dielectric and high surface energy is the most adequate device for detecting the shear force and applying texture recognition.

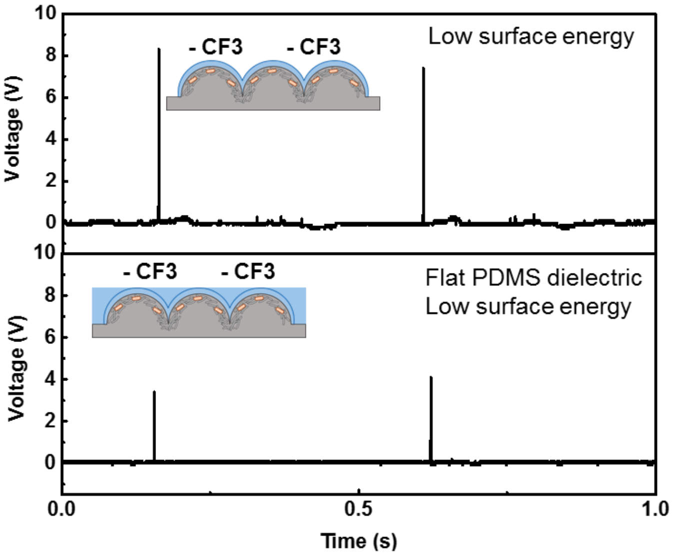

Figure S8. Voltage variations under dragging force with diverse types of E-skin devices.

III. 2. Preparation of various contacted objects for material/texture recognition application.

Various materials are tested to identify and classify the material and texture. Each sample were cut into 2.5 cm x 2.5 cm dimension and attached on slide glasses (2.5 cm x 2.5 cm) by using a double-sided tape except for human skin (**Figure S9** and **S10**). To investigate the detailed texture property variation, we prepared diverse morphologies of PDMS surface by modifying surface energy, roughness, and modulus. The surface energy of PDMS is easily controlled by UVO and CF_3-silane treatment [1]. As shown in **Figure S11**, roughness of PDMS is modulated by fabricating micropillar structure with a mold [5] and foam one with a sugar cube.[6] In addition, the mixing ratio of pre-polymer and crosslinking agent are chosen with 5:1, 10:1, 20:1, and 30:1, as modulus decreases and stickiness increases. The overall output electrical signals are varied with the material substance and surface properties (surface charge potential and adhesion force) of each material (**Figure S12- S14**).

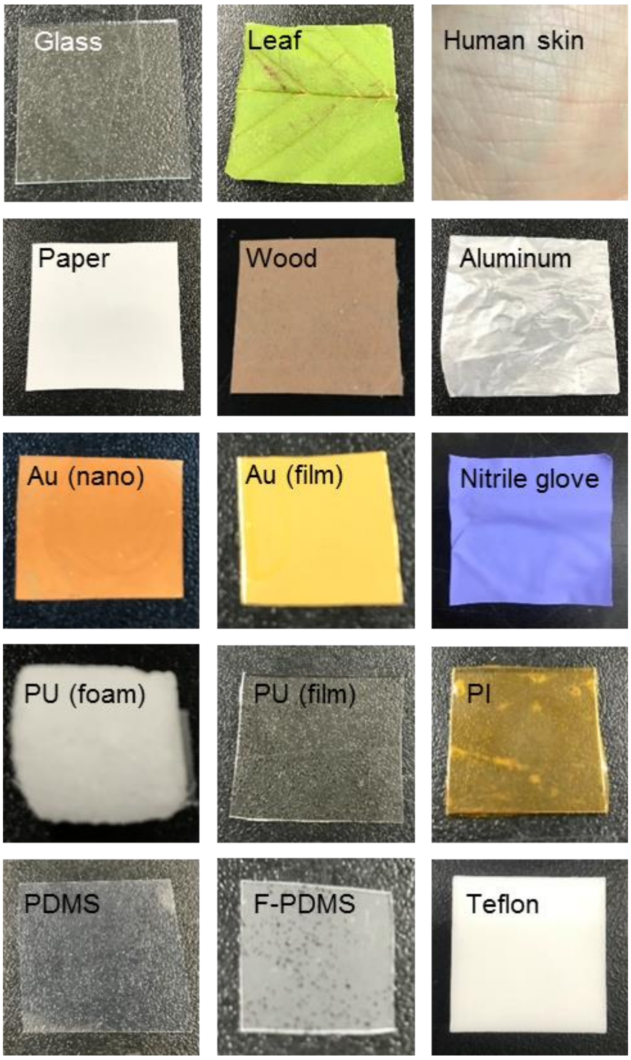

Figure S9. Various samples that can be detected and classified by our E-skin device.

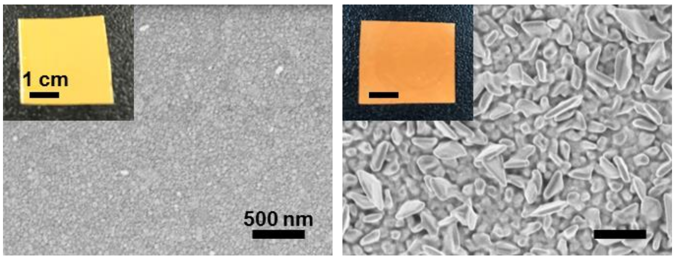

Figure S10. SEM images of surface morphology of gold film (left: gold film, right: nano-structured gold film (root mean squared surface roughness: 91.9 nm)). Insets are optical camera images of each material.

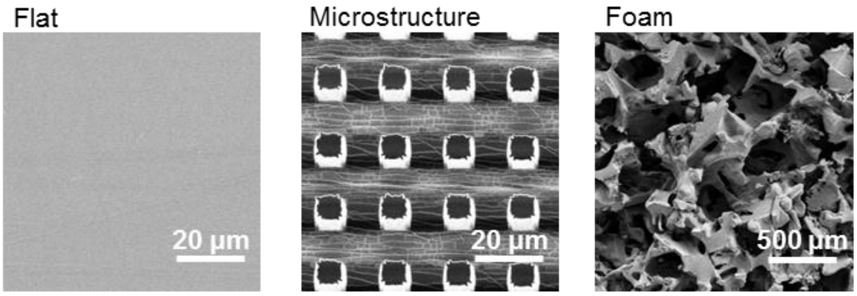

Figure S11. Various geometrical morphologies of PDMS.

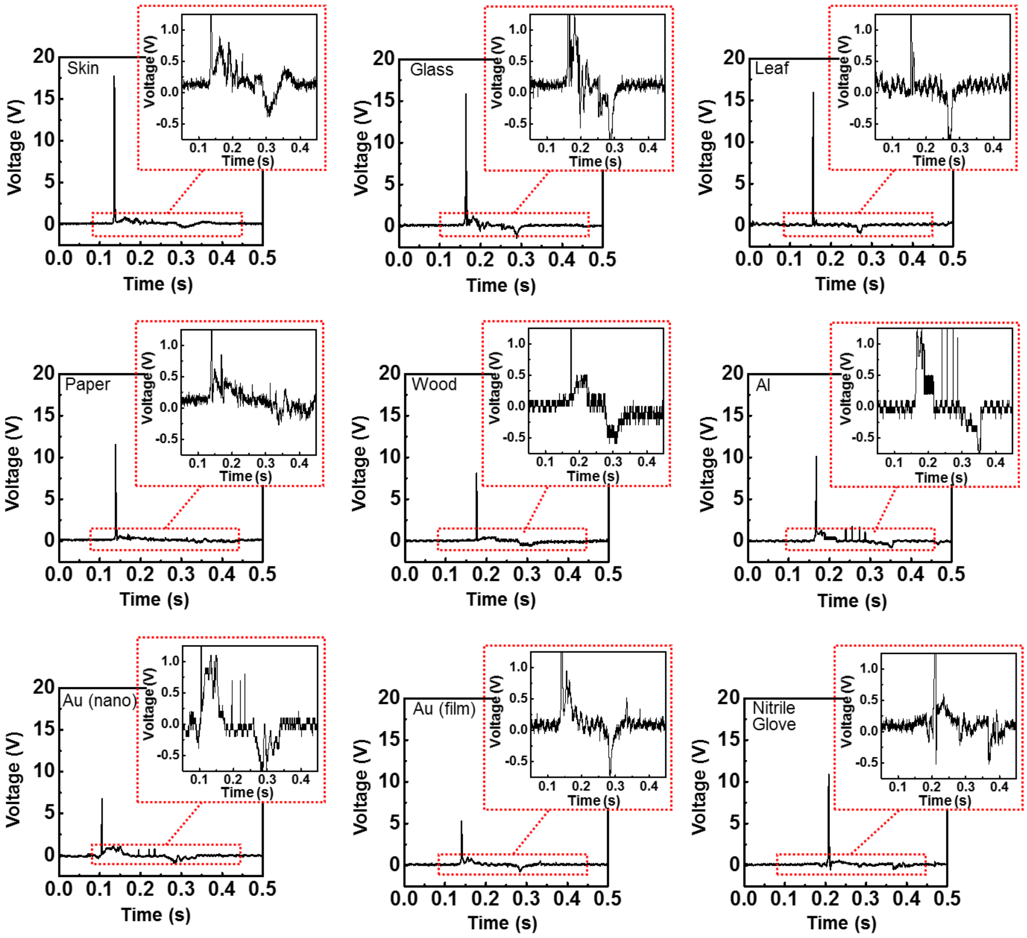

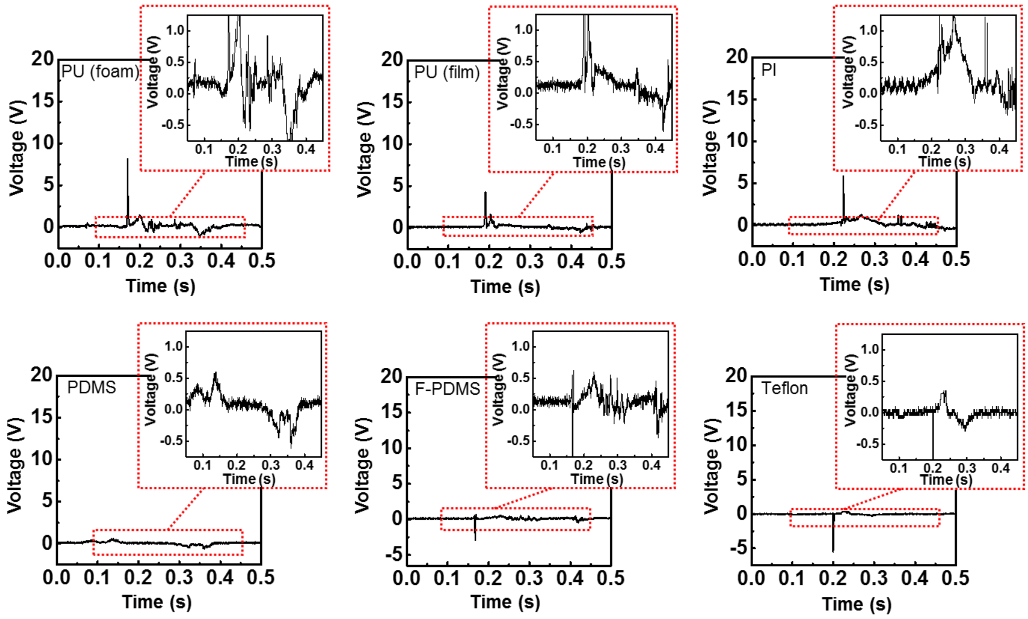

Figure S12. Voltage changes by dragging on each material with an E-skin

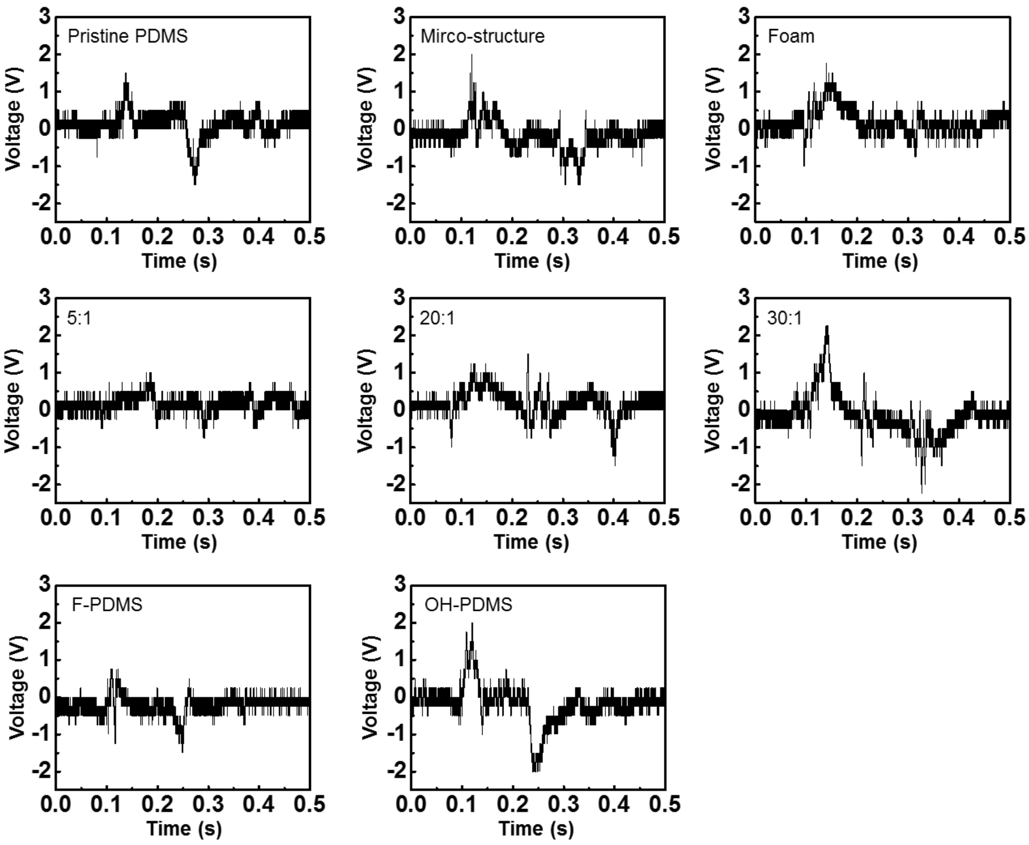

Figure S13. Voltage variations of various types of PDMS morphologies.

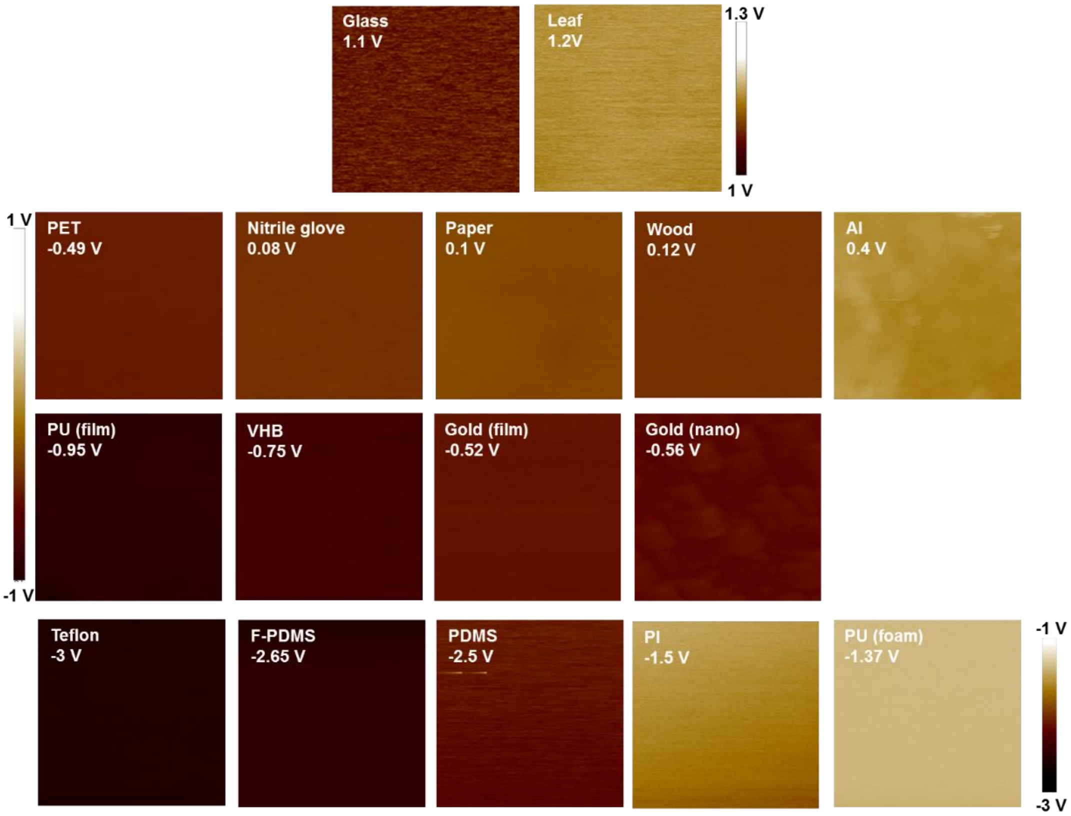

Figure S14. Surface charge potential distributions (3μm x 3μm) and the average value of contact potential differences (CPDs) of diverse material surfaces.

References

[1] G. Lee, S. G. Lee, Y. Chung, G. Y. Bae, S. Lee, S. Ryu, K. Cho, *Adv. Electron. Mater*. **2016**, *2*, 1600158.

[2] S. Chun, W. Son, H. Kim, S. K. Lim, C. Pang, C. Choi, *Nano Lett*. **2019**, *19*, 3305

[3] L. E. Stillwagon, R. G. Larson, G. N. Taylor, *J. Electrochem. Soc*. **1987**, *134*, 2030.

[4] M. Kwiatkowska, S. E. Franklin, C. P. Hendriks, K. Kwiatkowski, *Wear* **2009**, *267*, 1264.

[5] W. Liu, Z. Chen, G. Zhou, Y. Sun, H. R. Lee, C. Liu, H. Yao, Z. Bao, Y. Cui, *Adv. Mater.* **2016**, *28*, 3578

[6] W. G. Bae, D. Kim, M. K. Kwak, L. Ha, S. M. Kang, K. Y. Suh, *Adv. Healthcare Mater*. **2013**, *2*, 109
